# Trichomes and Allelochemicals in Tomato Genotypes Have Antagonistic Effects Upon Behavior and Biology of *Tetranychus urticae*

**DOI:** 10.3389/fpls.2018.01132

**Published:** 2018-08-14

**Authors:** João R. F. de Oliveira, Juliano T. V. de Resende, Wilson R. Maluf, Tiago Lucini, Renato B. de Lima Filho, Isabela P. de Lima, Cristiane Nardi

**Affiliations:** ^1^Horticulture Research Group, Department of Agronomy, Midwestern State University, Guarapuava, Brazil; ^2^Department of Agriculture, Federal University of Lavras, Lavras, Brazil; ^3^Laboratory of Entomology, Embrapa National Wheat Research Center, Passo Fundo, Brazil; ^4^Laboratory of Agricultural Entomology, Department of Agronomy, Midwestern State University, Guarapuava, Brazil

**Keywords:** *Solanum lycopersicum*, *Solanum habrochaites* var. *hirsutum*, zingiberene, glandular trichomes, biology, plant-resistance

## Abstract

Tomato genotypes selected for their high foliar zingiberene (ZGB) contents in a segregating F_2_ population were assessed to determine their effect on behavior and biology of *Tetranychus urticae* Koch, the putative resistance mechanisms involved and the role of trichomes on that resistance. Genotypes with contrasting ZGB content (RVTZ-09 = low ZGB, RVTZ-79 = high ZGB, RVTZ-142 = high ZGB, and RVTZ-331 = high ZGB) were selected from an interspecific cross between wild *S. habrochaites* var. *hirsutum* accession PI-127826 (high ZGB content and resistant to mites) and *S. lycopersicum* cv. Redenção (low ZGB content and susceptible to mites). To determine the effect of these genotypes on mite behavior and biology, free- and no-choice tests, as well as biological studies were performed. Types and densities of trichomes on the foliar surface and their correlation with ZGB contents was determined. Genotypes rich in ZGB (RVTZ-79, RVTZ-142, and RVTZ-331) presented a high number of types IV and VI glandular trichomes, and both type IV and VI densities were positively correlated with ZGB content. In the free-choice test, *T. urticae* showed a high preference toward *S. lycopersicum* cv. Redenção and the genotype RVTZ-09 (low ZGB content), whereas, genotypes with high ZBG content were less preferred. Moreover, on high ZGB genotypes, increase in the egg incubation period and in total mortality of nymphs, and decrease of fecundity rate were observed, indicating deleterious effects in mite biology. Results indicated that high ZGB/high glandular trichome densities genotypes present both non-preference and antibiosis mechanisms of resistance to the mite.

## Introduction

The two-spotted spider mite, *Tetranychus urticae* Koch (Acari: Tetranychidae), is a phytophagous and polyphagous mite species (Clotuche et al., 2011). In tomato, *Solanum lycopersicum* L. (formerly *Lycopersicon esculentum* Mill.), this arthropod causes severe damage on leaves and on fruits, especially when they are grown in greenhouses, where the mites find favorable environmental conditions (Meck et al., 2013). Considering the need for reduction of chemical applications (i.e., synthetic acaricides) for mite control, tomato breeding programs aimed to develop resistant cultivars should be considered an important contribution for the integrated management of this pest.

Studies have shown the potential use of some accessions of *S. lycopersicum* as resistance sources against arthropod pests. For instance, *S. lycopersicum* var. *cerasiforme* (Dunal), known as cherry tomato, has been studied for this purpose (Sánchez-Peña et al., 2006; Lucini et al., 2016). However, wild *Solanum* (section *Lycopersicon*) species are the most exploited as sources of resistance genes that may be deployed in tomato cultivars (Resende et al., 2009; Silva et al., 2009; Maluf et al., 2010; Oliveira et al., 2012; Dias et al., 2013; Lima et al., 2015).

Wild tomato accessions have been shown to affect behavior and biology of lepidopterans (Gurr and McGrath, 2001; Dias et al., 2013; Lima et al., 2015), coleopterans (Carter et al., 1989), hemipterans (Simmons et al., 2003; Resende et al., 2009) and mites *Tetranychus* spp. (Carter and Snyder, 1985; Gonçalves et al., 2006; Resende et al., 2008; Lucini et al., 2015). The main factor associated with resistance to pests in wild tomatoes, is reportedly the presence of glandular trichomes (especially, types IV and VI), which are responsible by storing and releasing allelochemical compounds (Maluf et al., 2001; Freitas et al., 2002; Simmons and Gurr, 2005). The zingiberene (ZGB) (a sesquiterpene) is an allelochemical stored and exuded by type IV and VI glandular trichomes present on plant surface of *Solanum habrochaites* Knapp and Spooner var. *hirsutum* Dunal (Maluf et al., 2001; Freitas et al., 2002; Gonçalves et al., 2006). The repellent effect of this wild tomato species against mites, imparted by presence of glandular trichomes containing ZGB, has been reported (Weston et al., 1989; Gonçalves et al., 2006).

Behavioral and biological bioassays are required to determine the mechanism(s) involved in the resistance against pests, in order to provide information on the extent of genotype resistance to herbivory. In this study, we selected tomato genotypes which contrasting ZGB contents in the F_2_ generation of the interspecific cross *S. lycopersicum* cv. Redenção ×*S. habrochaites* var. *hirsutum* accession PI-127826, and evaluated their effects on behavior and biology of the spider mite *T. urticae*, the putative resistance mechanisms involved (i.e., antixenosis and/or antibiosis), and the role of trichomes in the resistance.

## Materials and Methods

### Spider Mite Population Maintenance

Adults of *T. urticae* were field-collected at the experimental area of the Universidade Estadual do Centro Oeste do Paraná, Guarapuava, Brazil (25°23′S; 51°29W). Mites were taken to the Laboratory of Entomology where a colony was established under controlled conditions (25 ± 2°C, photoperiod of 12L:12D hours). Plants of *Canavalia ensiformis* (L.) (Fabaceae), grown in plastic pots (5L), were used as mite food source, and replaced when necessary. For use in the laboratory bioassays, a mite colony was established in a BOD incubator chamber at 25 ± 2°C; 70 ± 10% relative humidity, and photoperiod of 12L:12D hours.

The tomato genotypes used in bioassays were the acessions *S. lycopersicum* cv. Redenção (low ZGB content and susceptible to mites), *S. habrochaites* var. *hirsutum* accession PI-127826 (wild accession rich in ZGB and resistant to mites), and additional genotypes selected from the interspecific cross between PI-127826 × cv. Redenção.

These additional genotypes comprised one F_1_ plant (Redenção × PI 127826), and four plants selected from the F_2_ (Redenção × PI 127826) population, three of which (RVTZ-79, RVTZ-142, RVTZ-331) selected for high ZGB contents and one (RVTZ-09) selected for low ZGB.

The selected genotypes were cloned through rooting of axillary shoots in a tray filled with commercial substrate that was kept moist. Seedlings were transplanted into 10L plastic pots containing a mixture of commercial substrate: soil (1:1) and fertilized with NPK (04:14:08). The plants were kept in a greenhouse with daily irrigation. Expanded leaflets of 40/50-day-old plants were sampled and used in the laboratory bioassays.

### Zingiberene Contents and Trichomes Densities

In order to select for ZGB contents, 553 plants were analyzed [433 plants of the F_2_ generation (PI-127826 × cv. Redenção), 40 plants of the F_1_ generation, 40 plants of cv. Redenção and 40 plants of PI-127826]. The methodology proposed by Freitas et al. (2000) was used to quantify ZGB: six leaf disks (diameter 1 cm) were sampled from each plant, placed in tubes containing 2 ml of hexane, and then vortexed for 30 s. After that, the leaf disks were removed and the absorbance of the solution was measured using a spectrophotometer (Cary series – UV-Vis Spectrophotometer) at 270 nm of wavelength. The selection of genotypes was carried based on the absorbance values, which were above 0.700 for the three high ZGB genotypes, and below 0.250 for the low ZGB genotype.

Identification and quantification of trichomes on each genotype was based on images obtained using a scanning electron microscope (SEM) (Hitachi High-Tech TM3000 with tungsten filament, low vacuum and 15 kV). For this purpose, leaf disks (diameter 1 cm) taken from leaflets were inserted into the microscope. Luckwill’s (1943) and Toscano et al.’s (2001) classifications were followed to identify the trichome types.

Types IV and VI glandular trichomes, and type VIII non-glandular trichomes were counted separately. Counting was performed on both foliar surfaces (abaxial and adaxial) of four equal quadrants of each leaf disk, and numbers of trichomes per mm^2^ were recorded.

### Free- and No-Choice Tests

For free- and no-choice test, arenas (petri dishes with 6 cm diameter) were coated inside with a layer of household sponge topped with a layer of cotton-wool, both moistened in distilled water. In the free-choice test, leaf disks (diameter 3 cm) of the different genotypes evaluated were placed in pairs (one pair per arena) onto the cotton wool layer with abaxial side up. Leaf disks in each pair were connected each other using a transparent plastic coverslip (18 × 18 mm). After that, six 10-day-old adult *T. urticae* females collected from the colony were transferred into the center of the coverslip under a stereomicroscope (Nikon SMZ745T), allowing for free choice and mite access to both leaf disks. Because there were seven treatments, a full replication comprised 21 pairwise combination of genotypes (i.e., 21 arenas). Five full replications, totaling 105 petri dishes, were used, in a completely randomized design. After 24 h, the number of mites on each leaf disk were counted, and used to calculate the mite preference (%) for the respective genotype.

In the no-choice test, leaf disks (diameter 3 cm) of each genotype were placed separately onto the cotton wool layer, with abaxial side up, into the arenas. Six 10-day-old adult *T. urticae* female mites collected from the colony were transferred to each leaf disk under a stereomicroscope, and kept for 24 h. After that period, the number of eggs laid on each leaf disk was recorded. This test was conducted in a completely randomized design with 10 replications per genotype, comprising a total of 210 arenas. For both tests, the arenas were kept in a walk-in chamber at 25 ± 2°C; 70 ± 10% relative humidity, and photoperiod of 12L:12D hours.

In addition, another no-choice experiment was performed in order to evaluate the total distance traveled by the mite on leaflets using Weston and Snyder’s (1990) methodology. Leaflets (replications) from each genotype were sampled and fixed with abaxial side up on polystyrene sheets, using a metallic thumbtack (diameter 10 mm). Seven tomato genotypes (treatments) with 20 replications each were tested in a completely randomized design. Ten 6-day-old females were transferred onto each thumbtack, using a fine paint brush. After 10, 20, 40, and 60 min, the distances traveled by the females on the leaflet surface were recorded. The mean distances traveled by the mites after 60 min were determined for each genotype. This test is based on the assumption that lower distances covered by the mites indicate higher levels of mite repellence (e.g., negative effects on movement behavior).

### Biological Parameters of *Tetranychus urticae* on Tomato Genotypes

This study comprised two experiments. In the first, the incubation period and viability of the eggs, the duration and viability of young stages (larvae, protonymph, and deutonymph) and the longevity of adults were measured for the seven genotypes tested. Six leaf disks (diameter 2 cm) of each genotype, with abaxial side up, were equidistantly distributed into arenas (petri dishes, diameter 10 cm) coated inside with a layer of household sponge topped with a layer of cotton-wool, both moistened in distilled water. A 10-day-old *T. urticae* female + male pair was transferred and kept for 24 h onto each leaf disk; after that, the mites and eggs (except one), were carefully removed with a fine artist’s brush under a stereomicroscope, leaving one single egg per disk. Eggs, young stages and adults were daily observed to determine the parameters previously cited. Each set of six leaf disks within an arena was considered one replication. Altogether, 10 replications per treatment were used, in a randomized complete design.

In a second experiment, mite fecundity rate was evaluated. Ten leaf disks (diameter 2 cm) of a same tomato genotype, with abaxial side up, were equidistantly placed into plastic boxes (11 × 11 × 3.5 cm) coated inside with a layer of household sponge topped with a layer of cotton-wool, both moistened in distilled water. A 10-day-old *T. urticae* female was transferred onto each leaf disk, and was observed daily for 10 days to record the total number of eggs laid. During this period, the leaf disks were replaced by new disks of the same genotype every 2 days keeping the same female on the new disk.

Each plastic box with ten leaf disks of the same genotype, was considered a replication. Ten replications distributed in a completely randomized design were used for each of the seven genotypes tested.

Altogether, 60 and 100 observations (leaf disks) were taken per genotype respectively in the first and second experiments.

Both bioassays were carried out in a BOD incubator chamber at 25 ± 2°C; 70 ± 10% relative humidity, and photoperiod of 12L:12D.

### Statistical Analyses

Data were previously tested using Bartlett’s test to check for homogeneity of variance (*p* < 0.05), and then transformed to (x + 0.5)^1/2^ when necessary to fulfill the pre-requisites of analysis of variance (ANOVA). The mean number of trichomes and data related to behavioral and biological parameters of the mites were submitted to ANOVA, and treatment means were compared by Tukey test (*p* < 0.05). Data of *T. urticae* preferences (%) from the free-choice test were compared using Pearson’s Chi-Square test (χ^2^). The types and densities of trichomes and ZGB contents were submitted to Pearson’s correlation analysis and compared by the Student’s *t*-test using Microsoft Excel^®^ program. The biological and behavioral parameters evaluated were submitted to similarity grouping using the cluster analysis according to Linkage’s method. Statistical analyses were performed using the Sisvar^®^ program (Ferreira, 2011).

## Results

### Trichomes and Correlations With Zingiberene Content

**FIGURE 1:**
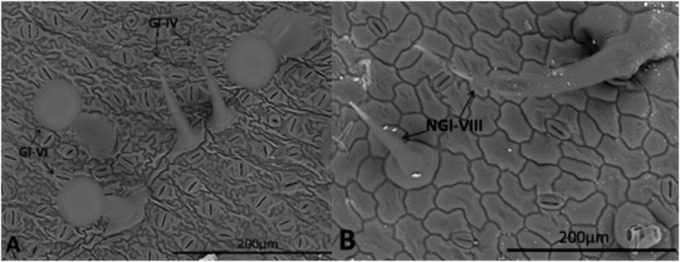
Leaflet surface of *S. habrochaites* var. *hirsutum* **(A)** and *S. lycopersicum* cv. Redenção **(B)** obtained in a scanning electron microscope (SEM) showing different trichome types. Type IV (Gl-IV) and VI (Gl-VI) glandular trichomes **(A)**, and type VIII (NGI-VIII) non-glandular trichome **(B)** according to Luckwill (1943) and Toscano et al. (2001) classification.

Densities of type VIII non-glandular trichomes and types IV/VI glandular trichomes varied among the genotypes in both abaxial and adaxial leaflet surfaces (**Table 1**). The highest non-glandular trichome density was observed in *S. lycopersicum* cv. Redenção, followed by the genotype RVTZ-09 (low ZGB content); in contrast, no non-glandular trichomes were found on wild accession (*S. habrochaites* var. *hirsutum* PI-127826) (**Table 1** and **Figures 1A,B**), and very low densities in the high ZGB genotypes.

**Table 1:** Zingiberene content (ZGB) and mean number (± SE) of glandular (types IV and VI) and non-glandular (type VIII) trichomes per mm^2^ present on abaxial (Ab) and on adaxial (Ad) surfaces of leaflets obtained from different tomato genotypes.

Genotype	ZGB content^1^	Glandular trichome type IV^2^			Glandular trichome type VI^2^			Non-glandular trichome type VIII^2^	Glandular types IV and VI (Ab + Ad)^2^
					
		Ab	Ad	Total	Ab	Ad	Total	Total	
*S. lycopersicum* (cv. Redenção)	0.084	0.0 ± 0.0d	0.0 ± 0.0d	0.0 ± 0.0d	0.0 ± 0.0d	0.0 ± 0.0d	0.0 ± 0.0d	84.5 ± 1.0a	0.0
*S. habrochaites* var. *hirsutum* (PI-127826)	1.099	20.7 ± 1.1bc	39 ± 2.7a	59.7 ± 1.4ab	23.2 ± 2.7a	4.0 ± 1.2ab	27.2 ± 3.0a	0.0	87.0 ± 4.0a
F1 plant (Redenção × PI-127826)	0.328	24.0 ± 1.3b	12.2 ± 0.9bc	36.2 ± 1.1c	6.0 ± 0.9b	4.5 ± 0.6a	10.5 ± 1.2bc	0.2 ± 0.2cd	46.7 ± 1.4b
RVTZ-09 (= Low)	0.247	8.0 ± 2.7c	4.7 ± 0.0c	12.7 ± 2.5d	0.0 ± 0.0d	1.5 ± 1.0ab	1.5 ± 0.9cd	13.5 ± 2.1b	14.2 ± 2.0c
RVTZ-79 (= High)	0.715	35.7 ± 6.2ab	8.2 ± 1.1b	44.0 ± 7.1bc	7.7 ± 1.1b	0.7 ± 0.5ab	8.5 ± 1.0bcd	5.7 ± 0.2c	52.5 ± 7.9b
RVTZ-142 (= High)	0.813	33.5 ± 6.5ab	16.5 ± 1.3b	50.0 ± 7.3bc	7.5 ± 1.3b	4.5 ± 2.9ab	12.0 ± 4.1b	3.5 ± 0.6cd	62.0 ± 4.7b
RVTZ-331 (= High)	0.746	51 ± 7.4a	35.2 ± 0.9a	86.2 ± 7.8a	1.5 ± 0.9c	2.7 ± 1.0ab	4.2 ± 1.6bcd	0.2 ± 0.2cd	90.5 ± 7.4a
Correlation coefficient with ZGB (r)		–	–	0.81^∗∗^	–	–	0.80^∗^	–0.66^ns^	0.90^∗∗^
CV (%)		17.5	16.9	11.8	16.6	35.5	20.6	9.1	9.4

*Means followed by the same lowercase letter within a column do not differ significantly by the Tukey test (p < 0.05).*

*^*ns*^Non-significant*,^∗^*and ^∗∗^Significant different by Student’s t-test (p < 0.05 and p < 0.01, respectively).*

*^*1*^Zingiberene content determined at 270 nm (see section Materials and Methods).*

*^*2*^Original data presented [for analysis, data were transformed in (x + 0.5)^*1/2*^].*

On the other hand, the wild accession PI-127826 and genotypes selected for high ZBG content (RVTZ-79, RVTZ-142, and RVTZ-331) presented high densities of types IV and VI glandular trichomes, on both surfaces, when compared to *S. lycopersicum* cv. Redenção (in which no glandular trichomes were observed) and the low ZGB genotype RVTZ-09 (**Table 1**). Densities of both types IV and VI glandular trichomes were significantly and positively correlated with ZGB content (*r* = 0.81, *p* < 0.01; and *r* = 0.80, *p* < 0.05, respectively) (**Table 1**).

### Free- and No-Choice Tests

**FIGURE 2:**
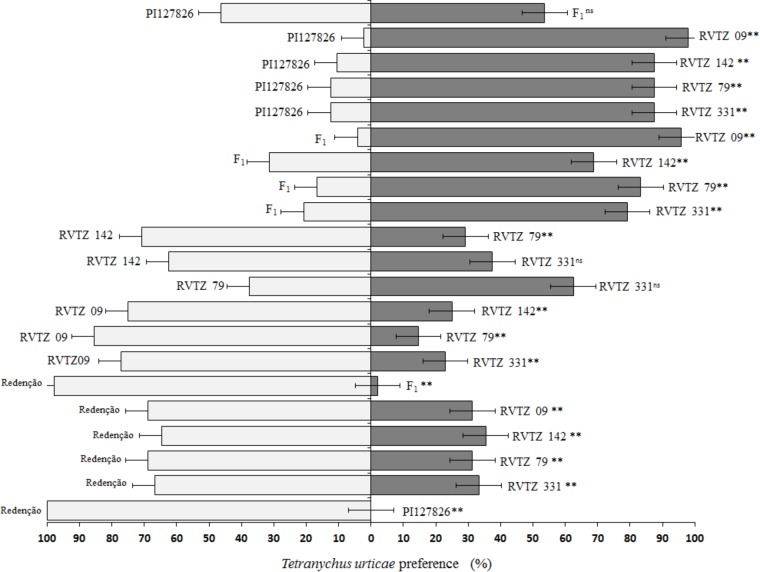
Preference ratios (%) of *Tetranychus urticae* on the 21 different pairwise combinations of tomato genotypes during the free-choice test. Redenção = *S. lycopersicum* cv. Redenção, PI127826 = *S. habrochaites* var. *hirsutum* accession PI-127826, F_1_ = F_1_ plant (PI-127826 × cv. Redenção), RVTZ-09 = RVTZ 2011-09 (low ZGB content), and RVTZ-79, RVTZ-142, and RVTZ-331 = RVTZ2011-79, RVTZ2011-142, and RVTZ2011-331, respectively (genotypes with high ZGB content). ^ns^ non-significant different, and ^∗∗^ significant different (*p* < 0.01) using Pearson’s Chi-Square test (χ^2^).

In the free-choice test, behavior of *T. urticae* females differed among the genotypes tested (**Figure 2**). Mites had significantly higher preference toward *S. lycopersicum* cv. Redenção, when paired with all other genotypes evaluated: over 60% of preference, reaching 100% when paired to the wild accession PI-177826. PI-177826 and F_1_ plant were significantly the least preferred (*p* < 0.01) by the mites at all genotype pairs assessed, even when compared to genotypes selected for high ZGB content. In addition, no statistical difference was observed between wild accession and F_1_ plant, indicating that F_1_ plant mite resistance level is similar to that of the resistant check treatment (**Figure 2**).

Genotypes selected for high ZGB content (RVTZ-79, RVTZ-142, and RVTZ-331) remained in an intermediary position between the least preferred genotypes PI-127826/F1 and the most preferred *S. lycopersicum* cv. Redenção/RVTZ-09 (low ZGB content). This latter genotype, showed similar responses to *S. lycopersicum* cv. Redenção when paired with genotypes rich in ZGB; therefore, it was also considered susceptible to the mite (**Figure 2**).

The no-choice test showed that the fecundity (eggs laid during 24 h) was reduced on genotypes with high ZGB content: (PI-127826, RVTZ-79, RVTZ-142, RVTZ-331, and F1) when compared to low-ZGB genotypes *S. lycopersicum* cv. Redenção and RVTZ-09. Fecundity in the high ZGB genotypes was ca. ≥ 4 times lower than in low ZGB genotypes (**Table 2**).

**Table 2:** Mean number (± SE) of eggs laid on leaf disks from different tomato genotypes during first 24 h of the no-choice test, and total distance traveled (mm) by the mites on the abaxial leaflet surface of the genotypes after 60 min.

Genotypes	Mean (± SE) of eggs laid^1^	Total distance traveled (mm)
*S. lycopersicum* (cv. Redenção)	19.1 ± 2.2a	35.0 ± 4.2a
*S. habrochaites* var. *hirsutum*	2.2 ± 1b	10.0 ± 2.1b
F_1_ (Redenção × PI-127826)	2.9 ± 0.6b	9.9 ± 2.3b
RVTZ-09 (= Low)	17.5 ± 1.7a	21.0 ± 5.3ab
RVTZ-79 (= High)	3.7 ± 0.9b	16.2 ± 2.5b
RVTZ-142 (= High)	3.4 ± 1.0b	18.2 ± 3.2b
RVTZ-331 (= High)	4.4 ± 1.3b	13.7 ± 1.9b
CV (%)	29.2	38.0
F	23.9^∗∗^	7.9^∗∗^

*Means followed by the same lowercase letter within a column do not differ significantly by the Tukey test (p < 0.05).*

*^∗∗^Significant different by F-test (p < 0.01).*

*^*1*^Original data presented [for analysis, data were transformed in (x + 0.5)^*1/2*^].*

Significant effects were observed on the movement behavior of the *T. urticae* released on the different tomato genotypes. Females released on leaflets of *S. lycopersicum* cv. Redenção were able to travel a significant higher distance (35 mm) after 60 min of evaluation than all other genotypes (<18 mm), except the low ZGB genotype RVTZ-09, which did not differ significantly from cv. Redenção (**Table 2**). The lower distances covered by the mite on genotypes with high ZGB content, was associated the presence of glandular trichomes (IV and VI), which were absent on *S. lycopersicum* cv. Redenção, and present at low density on RVTZ-09 (**Table 1**).

### Biological Parameters of *Tetranychus urticae* on Tomato Genotypes

The duration of egg incubation on genotypes with high ZGB content, RVTZ-142 (4.8 days), wild accession PI-127826 (4.5 days) and RVTZ-79 (4.3 days) tended to be significantly longer compared to genotypes with low ZGB content, (cv. Redenção and RVTZ-09). However, the viability of eggs in all genotypes evaluated was high (≥98%) (**Table 3**).

**Table 3:** Biological parameters [duration (days ± SE) of egg incubation and nymphal development time, egg and nymph viability (%), longevity of adults, and fecundity rate] of *Tetranychus urticae* kept on leaf disks of different tomato genotypes.

Genotype	Egg		Nymph		Adult	
	Incubation (days)^1^	Viability (%)	Duration (days)^1^	Viability (%)	Longevity (days)	No. eggs/female/day^1^
*S. lycopersicum* (cv. Redenção)	3.4 ± 0.1c	100	11.9 ± 0.2a	100	12.7 ± 0.5a	3.8 ± 0.2a
*S. habrochaites* var. *Hirsutum* (PI-127826)	4.5 ± 0.3ab	100	5.8 ± 0.3^2^b	0.0^3^	–	0.2 ± 0.1c
F_1_ plant (Redenção × PI-127826)	3.8 ± 0.2bc	100	6.1 ± 0.2^2^b	0.0^3^	–	0.2 ± 0.1c
RVTZ-09 (= Low)	3.4 ± 0.1c	100	11.8 ± 0.1a	100	12.0 ± 0.5a	3.7 ± 0.2a
RVTZ-79 (= High)	4.3 ± 0.2ab	100	5.5 ± 0.2^2^b	0.0^3^	–	0.6 ± 0.1b
RVTZ-142 (= High)	4.8 ± 0.2a	100	5.4 ± 0.3^2^b	0.0^3^	–	0.8 ± 0.2b
RVTZ-331 (= High)	3.8 ± 0.2bc	98.3	5.8 ± 0.2^2^b	0.0^3^	–	0.4 ± 0.1bc
CV (%)	32.6		17.6		31.1	14.9
F	9.9^∗∗^		298.7^∗∗^		–	99.0^∗∗^

*Means followed by the same lowercase letter within a column do not differ significantly by the Tukey test (p < 0.05), except for longevity of adults, in which was applied Student’s t-test (p < 0.05) to compare between S. lycopersicum cv. Redenção and RVTZ-09.*

*^∗∗^Significant different by F-test (p < 0.01).*

*^*1*^Original data presented [for analysis, data were transformed in (x + 0.5)^*1/2*^].*

*^*2*^Nymph development time up to 2nd stadium.*

*^*3*^Total mortality of nymphs in the 3rd stadium.*

The nymph stage was only completed in mites placed either on *S. lycopersicum* cv. Redenção or RVTZ-09; on both, all nymphs evaluated reach adulthood in a similar time (11.9 and 11.8 days, respectively), and adult longevities were also similar. In genotypes with high ZGB content (PI-127826, RVTZ-79, RVTZ-142, and RVTZ-331) and in F_1_ plants mites did not reach adulthood, because the mortality of the nymphs was total in the third instar. On those genotypes, nymphal stage had a short time span until death, ranging from 5.4 to 6.1 days – a span corresponding to the second nymphal stage (**Table 3**).

The fecundity rate was also adversely affected by the tomato genotypes with high ZGB content (fecundity rate from 0.2 to 0.8 eggs/female/day), whereas on *S. lycopersicum* cv. Redenção and RVTZ-09 the mites laid significantly more eggs (3.8 and 3.7 eggs/female/day, respectively) (**Table 3**).

### Cluster Analysis

**FIGURE 3:**
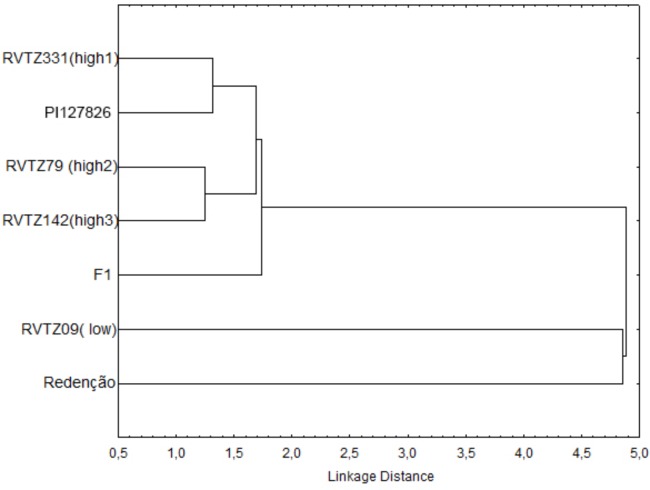
Relationships (dendrogram) between seven tomato genotypes with different contents of zingiberene using hierarchical cluster analysis (Linkage’s method). The cladogram has been based on number and type of trichomes, zingiberene content, and behavioral and biological parameters of *Tetranychus urticae.*

Cluster analysis of the tomato genotypes showed a clear contrasting relationship between the genotypes evaluated (**Figure 3**), and two clear groups were formed: one group comprised by *S. lycopersicum* cv. Redenção and RVTZ-09 (low ZGB content), and a second group formed by the genotypes with high ZGB content (RVTZ-79, RVTZ-142, RVTZ-331, and PI-127826) and the F1. Within-group differences between Redenção and RVTZ-09 could be at least partly explained by the slightly higher ZGB concentration in the latter (**Table 1**). However, ZGB content in RVTZ-09 is only slightly lower than in the F_1_ (**Table 1**), indicating that factors other than ZGB content in RVTZ-09 may account for its dissimilarity with Redenção.

All genotypes selected for high ZGB content might be considered close to the resistant check (wild accession PI-127826), as shown in the cluster analysis (**Figure 3**). The genotypes selected for high ZGB content formed two distinctive pairs strongly related each other __ first pair composed by RVTZ-331 and PI-127826, and the second one by RVTZ-79 + RVTZ-142.

The presence of glandular trichomes on genotypes selected for high ZGB content (**Table 1**) was strongly and negatively associated with the behavioral and biological parameters of the mite, except with egg incubation period (**Table 3**), which was the parameter less affected by the genotypes rich in ZGB. Thus, the results indicate that the presence of glandular trichomes lead to negative effects on mite behavior and biology.

## Discussion

Plants that produce and release chemical compounds (allelochemicals) affecting arthropod behavior and biology, may express resistance through both antixenosis (non-preference) and/or antibiosis (War et al., 2012). In tomato, resistance has been extensively reported in the literature as mediated by different allelochemicals against arthropod pests such as mites (Gonçalves et al., 2006; Resende et al., 2008; Lucini et al., 2015; Lima et al., 2016), whiteflies (Freitas et al., 2002; Silva et al., 2009; Lima et al., 2016), and lepidopterans (Maluf et al., 2010; Dias et al., 2013; Lima et al., 2015).

In the behavioral test (free-choice test), our results indicated lower preference of *T. urticae* toward the wild ZGB-rich genotype (PI-127826), followed by the F_1_ plant and genotypes selected for high ZGB content (RVTZ-79, RVTZ-142, and RVTZ-331). In contrast, *S. lycopersicum* cv. Redenção and RVTZ-09 (low ZGB content) were the most preferred genotypes by *T. urticae*. Lima et al. (2016) evaluating these same ZGB-rich genotypes observed a strong repellency effect against *T. urticae* and whitefly *Bemisia tabaci* (Genn.). The results indicated that non-preference is a mechanism by which ZGB-rich genotypes express resistance to the two-spotted spider mite.

Nonetheless, the genotypes rich in ZGB also presented the antibiosis type of resistance, indicated by their deleterious effects on biological parameters of *T. urticae*. In general, with ZGB-rich genotypes there was an increase of the egg incubation period, total mortality of nymphs, and a strong decrease in the fecundity rate. The adverse effect of tomato genotypes with high allelochemical contents on arthropod biology parameters is also documented in the literature for several species (Eigenbrode and Trumble, 1993; Azevedo et al., 2003; Fancelli et al., 2005; Moreira et al., 2009; Silva et al., 2013). Evaluating tomato genotypes with high content of the allelochemical acylsugar, Lucini et al. (2015) observed that they also presented both mechanisms non-preference and antibiosis against *T. urticae*, a situation analogous to our current findings for ZGB-rich genotypes. No similar reports on the deleterious effect of high-ZGB tomato genotypes on *T. urticae* mite survival, fecundity and longevity could be found in the literature.

The high density of glandular trichomes and its associated high ZGB content were therefore responsible for adverse effects upon both the behavior and the biology of *T. urticae* mites, indicating that both resistance mechanisms, non-preference and antibiosis, are present. In addition, *S. lycopersicum* cv. Redenção presented a high number of non-glandular trichomes, which did not cause negative effects on mite behavior and biology.

Other studies have demonstrated the effect of glandular trichomes present on *S. habrochaites* var. *hirsutum* on *Tetranychus* spp. (Carter and Snyder, 1985; Maluf et al., 2001; Freitas et al., 2002). Several other studies have also reported negative effects on behavior and biology of arthropod pests associated with glandular trichomes present in wild tomato accessions and in genotypes selected for high allelochemical contents (Freitas et al., 2002; Maluf et al., 2007; Alba et al., 2009; Lucini et al., 2015).

In this study, we demonstrated that ZGB content was positively correlated with both types of glandular trichomes (IV and VI), although the density of type IV was much higher than that type VI trichomes. These results agree with reports in the literature (Freitas et al., 2002; Gonçalves et al., 2006). Allelochemicals other than ZGB have been associated with glandular trichomes; e.g., acylsugar stored in type IV glandular trichomes of *Solanum pennellii* Correll accession LA-716 (Lucini et al., 2015) and *Solanum pimpinellifolium* accession TO-937 (Alba et al., 2009).

The results support that genotypes selected for high ZGB content (RVTZ-79, RVTZ-142, and RVTZ-331) are potential sources of resistant genes against *T. urticae* mites and possibly against other pests, in tomato breeding programs.

## Author Contributions

JdO designed and performed the experiments, carried out the statistical analyses, and wrote the project and the manuscript with support of TL and CN. JdR and WM devised the project, the main conceptual ideas and provided the plants and laboratory apparatus. TL and CN contributed to the design and implementation of the research, to the analysis of the results, and to the writing of the manuscript. CN were involved in planning and supervised the work. RdLF and IdL performed the plants and acari experiments with support of JdO and contributed in discussion of the manuscript in consultation with JdO. All authors provided critical feedback, helped in discussion, and contributed to the final manuscript.

## Conflict of Interest Statement

The authors declare that the research was conducted in the absence of any commercial or financial relationships that could be construed as a potential conflict of interest. The reviewer GDS and handling Editor declared their shared affiliation at time of review.
